# Intestinal congestion and reperfusion injury: damage caused to the intestinal tract and distal organs

**DOI:** 10.1042/BSR20211560

**Published:** 2021-09-06

**Authors:** Yajing Chen, Weigao Pu, Ewetse Paul Maswikiti, Pengxian Tao, Xuemei Li, Dengfeng Wang, Baohong Gu, Yang Yu, Lei Gao, Chengji Zhao, Hao Chen

**Affiliations:** 1 Department of Tumor Surgery, Lanzhou University Second Hospital, Lanzhou, China; 2 Department of Pediatric Surgery, Lanzhou University Second Hospital, Lanzhou, China; 3 The Second Clinical Medical College, Lanzhou University, Lanzhou, China; 4 Key Laboratory of Digestive System Tumors of Gansu Province, Lanzhou, Gansu, China

**Keywords:** bacterial translocation, Congestion-reperfusion injury, inflammatory factors, intestinal barrier, microcirculation

## Abstract

In clinical practice, intestinal autologous diseases, ailments and organ transplants can cause severe congestive damage to the intestinal tract. However, after the etiological factor is gotten rid of and blood flow is free without any hinderance, further damage to the intestinal wall often occurs, causing other related organ dysfunctions. This ultimately results in intestinal congestion reperfusion injury (ICRI). When the structure and function of the intestine are destroyed, bacteria, metabolites and endotoxins in the intestinal tract perfuse and enter the portal vein through the already compromised intestinal mucosa, to the other organs via the liver. Nevertheless, this gives rise to further aggravation of the injury, and reperfusion injury syndrome occurs. ICRI is a very common complication encountered by clinicians, and its harm is more severe and serious as compared with that caused by ischemia–reperfusion. Quite a few number of studies on ICRI have been reported to date. The exact mechanism of the injury is still idiopathic, and effective treatment strategies are still limited. Based on recent studies, this article is aimed at reviewing the destruction, damage mechanisms resulting from ICRI to the intestinal anatomical sites and distant organs. It is geared towards providing new ideas for the prevention and therapeutic approaches of ICRI.

## Introduction

Congestion is termed a passive process that occurs systematically and locally in different anatomical sites of the human body system. The intestinal sites are core organs for stress responses [1]. As such, they are also one of the most sensitive and most prone organs for congestion and intestinal congestion reperfusion injury (ICRI). Common clinical diseases such as liver transplantation, due to hepatic cellular carcinomas, portal vein occlusion, intestinal strangulation, intestinal malrotation and obstruction, inflammatory bowel diseases (IBDs), necrotizing enterocolitis, and mesenteric vein thrombosis may result from ICRI progression [2–5]. Studies have found that compared with intestinal ischemia, intestinal congestion is more likely to cause severe tissue damage, and recovery is very slow. Furthermore, its degree heightens with increasing congestion time [6]. Moreover, this is because the hemodynamic changes of the two disease processes are different. Although, both ischemia and congestion cause tissue ischemia and hypoxia, there are additional tensional and metabolite accumulations in the capillaries of tissues and organs during congestion. Intestinal mucosal damage and destruction occurs at an earlier stage and the damage mechanism is more complicated. When blood flow resumes reperfusion; congestion returns more slowly than ischemic blood flow, so the destruction caused by congestion to tissues is more severe than that given rise to by ischemia [7,8].

The intestinal site is the body’s main reservoir for bacteria. Moreover, it forms an indispensable barrier to harmful substances and pathogens from the external environment [9,10]. Intestinal congestion and reperfusion injury increase intestinal permeability, and results in the impairment of the intestinal barrier. Intestinal bacteria, endotoxin and other undifferentiated products become displaced in the process. In severe cases, it induces the cascade of inflammatory mediators to expand, causing a catastrophic damage to various organs throughout the body which are life-threatening [2,11]. ICRI’s disease manifestations and prognosis have been studied and confirmed clinically. Furthermore, its underlying mechanisms have been preliminarily studied, clinicians and medical researchers have paid attention to its phenomenon. Therefore, this article is aimed at discussing some related characteristics and mechanisms resulting from ICRI.

## ICRI and intestinal mucosal barrier

The intestines are the main organs that absorb nutrients. Therefore, the intestinal mucosal barrier is the first parclose between the intestines and the external environment. It is also a barrier to protect the internal environment within the human body. It is also a vital organ in reducing the invasion of pathogens and absorption of toxins.

The composition of the intestinal mucosal barrier consists of four parts, mechanical, chemical, immune, and biological barriers [10,12]. The mechanical barrier consists of intact epithelial cells, intercellular connective complexes of which tight junctions are major features mucous layers and biofilms. Tight connections among cells close the gap between adjacent intestinal epithelial cells, prevent bacteria and antigens in the intestinal cavity from entering the intestinal lamina propria to activate immune cells, and also inhibit the occurrence of abnormal mucosal immune responses [13]. Chemical barriers consist of digestive enzymes, lysozymes, mucopolysaccharides, and glycolipids secreted from the gastrointestinal lining. Furthermore, the composition of bacteriostatic substances produced by normal parasitic bacteria, has bactericidal activities, lytic, inhibition and invasion by pathogenic bacterial processes. Meanwhile, the intestinal mucus layer is mainly composed of mucin (MUC) secreted by intestinal goblet cells and some studies have shown that glycosylation of the intestinal mucin O-glycan is an essential process for the synthesis of MUC2 with an advanced activity of the barrier [14]. Among these functions, one of them is to maintain the function of the intestinal mucosal barrier [15]. Nonetheless, the immune barrier is mainly composed of gut-associated lymphoid tissues (GALTs) and the secretion of immunoglobulin (sIgA). GALT’s major role is to maintain the integrity of the intestinal mucosal barrier, and sIgA is the first line of defense against the adhesion and colonization of pathogenic bacteria along the intestinal mucosa [16]. The biological barrier is essentially an interdependent and interacting microecosystem composed of the intestinal resident flora. It is mainly composed of anaerobic bacteria, facultative anaerobic bacteria, and aerobic bacteria and approximately 99% of them are obligate anaerobic bacteria, which are the most dominant in the intestinal flora. It has some antibacterial and regulatory functions with regard to the intestinal immunity [17].

Under normal circumstances, the intestinal barrier allows only small numbers of antigens and bacteria to pass through the mucosa and to protect the underlying immune cells. Injury and dysfunction to any of the above structures will affect the function of the intestinal barrier system [12]. In addition to the occurrence and progression of ICRI, intestinal mucosal blood stagnation and hypoxia, the generation of reactive oxygen species (ROS), instantly attaches to intestinal epithelial cells. Henceforth, large accumulation of ROS can damage all biologically active substances such as nucleic acids and proteins. This results in the causation of lipid peroxidation of biological membranes, non-peroxidative mitochondrial damage, DNA destruction, and protein cross-linking [2,18]. Thereby, changing the cell biofilm and cellular structure, causing apoptosis and necrosis. In addition, ROS can directly oxidize and damage the intestinal mucosal mechanical barrier, change its permeability, and then invade toxic substances as well as pathogens in the intestinal site. Interference with the stability of the environment in the tissue, induction of inflammatory and immune responses end-up resulting in the production of a series of clinical ailments and disorders such as enterogenic shock, sepsis, and even multiple organ failures [19].

Presently, related researches on ICRI have not discussed in detail and in-depth of the pathophysiological changes and corresponding mechanisms of the above-mentioned four barriers cause to intestinal mucosal destruction. Based on the integrity and functional complexity of the intestinal barrier structure, congestion reperfusion injury can be established in subsequent studies to explore the changes in pathology, cytokines, and other barrier mechanisms.

## ICRI and bacterial translocation

The whole human gut microbiome consists of approximately 1150 bacterial species, with each individual host having roughly 160 species. Different and various intestinal microorganisms constitute, grow, and multiply in extremely complex intestinal microecosystems. The symbiotic interaction between bacterial groups play major roles in the maintenance of the intestinal homeostasis [9]. Furthermore, the main functions of the intestinal flora include metabolic activities, nutritional effects, immunity, and protection of the host from the invasion of foreign microorganisms. Bacterial translocation is therefore a phenomenon and a rare occurrence in which normal intestinal organisms move to various sterile tissues and organs outside the intestine through increased intestinal permeability due to the barrier of the intestinal destruction [20,21]. Moreover, bacterial translocation comes mainly as a result due to an impaired, weakened, and destroyed intestinal mucosal barrier. In fact, bacteria do not require crossing the intestinal epithelial barrier to trigger sepsis, instead they just need to be stationed at one place to induce sepsis. Inflammatory substances produced by the intestinal wall or toxic substances produced from the intestinal tract cause systemic damage through displacement. Therefore, the term bacterial translocation includes not only the translocation of live intact bacteria, but also toxins, antigens, or other microbial products from the intestinal cavity into the circulation leading to systemic inflammation and various diseases.

As mentioned earlier, ICRI causes damage and much destruction to the normal intestinal barrier and floral morphology, causing pathogenic bacteria to multiply and duplicate. Henceforth, a large amount of endotoxin is released into the bloodstream. The influx of pathogenic bacteria and endotoxins into the bloodstream excessively activates the immune response, causing the release of limitless inflammatory mediators, further aggravating the intestinal mucosal damage [20,22]. Possible mechanisms from the above changes include and are not limited to, the intestinal villi ischemia and hypoxia after intestinal congestion with oxygen free radicals, tumor necrosis factor (TNF)-α, and interleukin (IL) 1 (IL-1). These mediate the destruction of tight junctions between intestinal tissue cells such as *Helicobacter pylori* toxin, *Clostridium difficile*, *Bacillus* toxins A and B. Thereafter, some bacterial toxins affect intestinal epithelial permeability without causing structural changes in tight junctions [23,24]. Lastly, the occurrence of intestinal inflammation releases IL-1β which damages the intestinal epithelial cells. Post-reperfusion, a large amount of oxygen free radicals in the blood further aggravates the intestinal mucosa and increases the permeability of the intestinal mucosa. Intestinal congestion and ischemia as well as hypoxia reduce the intestinal mucosal secretion which functions and increases the adhesion of bacteria on the intestinal mucosal lining. Furthermore, bacterial overgrowth and colonization in the intestinal cavity, bacterial toxins and proteolytic enzymes damage the intestinal mucosal barrier and leads to the intestinal bacterial translocation process. The secretion of IgA synthesis is reduced, immune barrier function is also reduced, and endotoxemia results in systemic specificity and ultimately the decline of non-specific immune function promotes bacterial translocation [2,20].

Changes in four components of the intestinal barrier in ICRI are not independent of each other, they affect one another to form a complex network of interactions. For instance, transformations in immune cellular function can change and cause some alterations in the intestinal flora. These changes in the intestinal flora can temper with the secretion of intestinal secretions, and changes in secretions further affect the intestinal flora homeostatic environment. Therefore, the study of bacterial translocation should be scrutinized from different aspects.

## ICRI and immune system inflammatory responses

There are a variety of immune cells throughout the gut, and GALT is probably the most recognizable part of the intestinal immune system. Cytokines and chemokines are key soluble protein mediators for intercellular communication and maintain intestinal mucosal homeostasis, but may also be key leading factors of intestinal inflammation and inflammation-related damages.They can positively or negatively affect the integrity of the intestinal epithelial barrier, alter intestinal permeability, and induce or inhibit the proliferation and death of intestinal epithelial cells through innate or adaptive immune cells, infiltrating inflammatory cells, or the intestinal epithelial cells themselves [25]. ILs, for example, IL-10 or IL-2 missing genes cause spontaneous colitis in mice, indicating that these factors are necessary for the colon’s dynamic state, and other cytokines, including the release of IL-6, TNF, IL-18, IL-1β and IL-17, excessive expression in inflammatory bowel. Moreover, this is considered to be one of the leading to intestinal injury [26].

Post-ICRI, the intestinal barrier dysfunction causes intestine-derived bacteria/endotoxin translocation, which initiates the release of inflammatory mediators and cytokines, and triggers systemic inflammatory responses. Among these, TNF-α plays a central role in complex cytokine chain reactions, induces polymorphonuclear neutrophils (PMNs) to aggregate and adhere in tissues finally releasing IL- 6 and inflammatory mediators such as IL-8, which end up causing damage to the intestinal mucosal barrier resulting from a variety of diseases [27]. TNF-α is mainly secreted by activated monocytes and macrophages, and it has regulatory cytokines involved in host anti-infection, anti-tumor immunity, and other physiological functions. Studies have shown and found that the direct toxic effects of TNF-α include and are not limited to; (1) direct dismantling of the intestinal mucosa, resulting in increased intestinal barrier permeability; (2) promotion of PMN release of free radicals, causing microcirculation disorders; (3) activation of the complement system, mediation. The production of lipid mediators and other peptide mediators which cause cell disruption [28]. This may result in major causes of the intestinal wall destruction, specifically to the mechanical barrier. In addition, there are other relevant cytokine changes such as: (1) Caspase3 elevation. Caspase3 activates the apoptosis pathway leading to apoptosis of the cells along the intestine, which may be an intrinsic mechanism of impaired intestinal function after ICRI [29]. (2) Increased myeloperoxidase (MPO) activity. MPO is a relatively stable enzyme in neutrophils. When PMN aggregates and adheres, its level can reflect the degree of neutrophil infiltration and inflammation in tissues [2]. (3) MDA is also elevated in the process. MDA is a metabolite of lipid peroxidation. Studies have shown and found that the levels of malondialdehyde in the intestinal and liver tissues are increased after ICRI, and the increase is consistent with the histopathological injury score, suggesting a close relationship between malondialdehyde and tissue damage [18]. (4) Increased diamine oxidase (DAO) activity. Nevertheless, under normal circumstances, DAO is basically not detected in blood circulation. When the intestinal mucosal villi epithelium is damaged and intestinal barrier dysfunctions, DAO level is released in serum, which then reflects the intestinal mucosal mechanical barrier’s functionality [2]. (5) Increased d-lactic acid level. d-Lactic acid is a metabolite of various bacteria in the intestines. The intestinal barrier is damaged when intestinal congestion occurs, and the bacteria in the intestinal cavity multiplies and duplicates. d-lactic acid production increases and enters blood circulation. There is no catabolic effect on d-lactic acid, so the measurement of d-lactic acid in blood reflects the barrier function of the intestinal mucosa, indicating that it has acrucial and essential value in predicting intestinal mucosal barrier destruction [30].

## ICRI and apoptosis

ICRI-induced intestinal mucosal epithelial damage and destruction have the likelihood of ending up resulting in an apoptotic process. Cell apoptosis is a kind of programmed cell death (PCD). PCD includes various modes of cellular extinction or death. Among these, apoptosis was the first mode and cellular death process researchers first comprehended. In recent years, researchers have also found special programmed inflammatory death forms such as pyroptosis and necroptosis. Meanwhile, apoptosis is mostly accomplished and associated with the caspase family [31]. Caspase, performs two main functions: inducing apoptosis and participating in inflammatory responses. Furthermore, it is known that caspase-2, -3, -6, -7, -8, -9, and -10 are involved in the apoptotic signaling process, while caspase-1 and -11 are mainly involved in cytokine activation and inflammatory responses [32]. Generally speaking, caspase-8 and -10 mediate apoptosis of the death receptor pathway and are recruited to Fas and TNF-α cell surface death receptor 1 (TNFR1) death receptor complexes, respectively, while caspase-9 is involved in apoptosis of mitochondrial pathway and is recruited to apoptosome composed of Cyt *c*/*d* ATP/apaf-1 [33].

With regard to ICRI, due to intestinal mucosal barrier damage and inflammatory response, inflammation-related factors are released into the blood circulation, apoptosis is initiated, and cell death is induced. However, existing experimental studies have shown that venous congestion and either congestion reperfusion can induce the rat model’s small intestinal mucosal apoptosis and TNF-α synthesis, and part of apoptosis is mediated by TNF-αI type cell death rather than through the induction of mitochondrial cytochrome *c*-mediated II type of cell death [27]. TNF-α is a multidirectional cytokine that, in addition to its role in infection and immunity, can induce apoptosis in multiple cell types. TNFR1 leads to the accumulation of TNF receptor 1-associated with death domain (TRADD), which allows procaspase-8 to initiate and induce death signals [34]. When cells are exposed to apoptosis-inducing signals, apoptotic execution caspases (caspase-3, -6, and -7) are activated, resulting in DNA fragmentation and further formation of apoptosis. Currently, there are few studies on apoptosis in ICRI, and further studies on its mechanism are needed and still are to be explored.

## ICRI and distant surrounding peripheral organs

**Figure 1:**
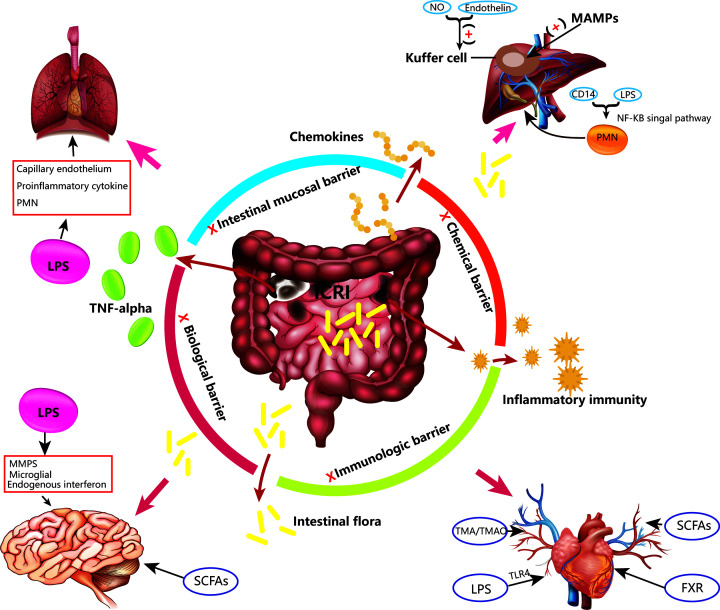
Effects of ICRI on the intestine and distal organs When ICRI occurs, four of the gut barriers; mechanical, chemical, immunity, and/or biological are dismantled and various inflammatory mediators, cytokines, endotoxins, and related bacterial metabolites are produced that cause damage to the intestine itself. In addition, these substances enter the distal organs with blood circulation or other routes, causing damage to the distal organs. Among them, endotoxins have the most voluminous effect. It can induce the production of downstream products of inflammation by combining with TLR4 and increase the degree of heart failure; and can also stimulate PMN to further intensify the inflammatory response and trigger the cells to aggravate liver damage; or increase the matrix metalloproteinases. The production of plastid cells destroys the blood–brain barrier and the central nervous system situation; it also causes damage to pulmonary blood vessels and tissues. Abbreviations: FXR, farnesoid X receptor; LPS, lipopolysaccharide; MAMP, microbe-associated molecular pattern; SCFA, short-chain fatty acid; TLR4, toll-like receptor 4; TMA, trimethylamine; TMAO, trimethylamine N-oxidation.

The initial damage of intestinal congestion reperfusion is located in the intestinal mucosa, and its effect on distant organs and even the whole body is due to the destruction of the intestinal mucosal barrier during congestion, accumulation of intestinal bacteria, metabolites, and endotoxins [9]. Blood flow is restored at a later stage and as such, these substances enter the liver, blood system or transferred from the lymphatic system to other organs along with portal venous blood flow, resulting in reperfusion injury syndrome causation [2] (Figure 1).

### ICRI and the liver (hepatic organ)

ICRI will inevitably be encountered in various clinical scenarios such as in liver transplantations and liver tumor surgical procedures. This is an important risk factor leading to poor surgical outcomes leading to increased rates of morbidity and mortality. Many studies have shown that intestinal endotoxin translocation and the degree of translocation are closely related to the prognosis of liver transplantation and liver resection. Intestinal congestion and reperfusion can increase liver ischemia–reperfusion injury [8,20,35]. Following, intestinal congestion reperfusion, bacteria, metabolites, and endotoxins reach the liver through the portal venous system. First and foremost, they activate Kupffer cells in the liver and cause them to secrete pro-inflammatory factors such as TNF-a, IL-1, and IL-6. Simultaneously, it increases the expression of adhesion molecules, promotes neutrophil chemotaxis, aggregation, and adsorption. A large number of inflammatory factors are released, leading to a ‘waterfall-like’ inflammatory response, acting on hepatic sinusoidal endothelial cells, and causing microcirculation disorders. Liver cells are hard hit by necrotic apoptosis, which is manifested by increased liver enzymes and bilirubin content in the blood [36]. The intestinal bacteria escape from the stable microbe-associated molecular patterns (MAMPs) in the intestinal cavity, and when they reach the liver, they are combined with the pattern recognition receptors of Kupffer cells to make the number of these cells in the liver increase. Gut-derived peptidoglycan activates the body’s innate immunity, and the level of gut-derived MAMPs is closely related to the number, functional activity, and maturity of Kupffer cells in the liver [37]. CD14 of Kupffer cells is a receptor for endotoxin lipopolysaccharide (LPS). Furthermore, post-binding to LPS, it activates the NF-KB signaling pathway downstream of toll-like receptor 4 (TLR4), induces the synthesis of pro-inflammatory factors and adhesion molecules, causing oxygen free radicals, protease production, and neutrophil activation which eventually aggregates, finally leading to hepatocyte damage [38]. In addition, the imbalance of endothelin and nitric oxide levels narrows the hepatic sinusoidal lumen, vasoconstriction, and cell adhesion lead to liver microcirculation disorders, induce severe and long-term liver hypoxia, and necrosis. This then brings about Kupffer cell activation.

The inflammatory factors and related metabolites produced after liver injury by various causes pass through the intestinal–hepatic circulation of the bile and damage the intestinal mucosa after reaching the intestine [24,33]. Meanwhile, some of them reach the intestine through systemic blood circulation to further enhance intestinal congestion and reperfusion injury. Therefore, the impact of ICRI on the liver cannot be underestimated and underrated. It has some close effects related to the prognosis and disease progress of patients with liver ailments. Exploring the exact pathogenesis is of great significance for the prevention and treatment of liver injury.

### ICRI and the heart (cardiac organ)

ICRI impairs and diminishes the integrity of the intestinal barrier, leading to increased intestinal permeability, endotoxins, and related inflammatory factors entering the bloodstream, and bacterial translocation. Henceforth, the catastrophic and disruption of the intestinal barrier allows LPS to enter blood circulation. LPS is a classic pathogen-associated molecular model that induces the expression of multiple downstream products of inflammation through the TLR4 pattern recognition receptor [39]. Studies have shown that intestinal absorption of endotoxin is an important stimulus for the increase in systemic inflammatory cytokines during heart failure (HF) [40,41]. Meanwhile, microorganisms of the gastrointestinal tract function as endocrine organs by producing bioactive metabolites, which directly or indirectly, affect the host’s physiological functions. Intestinal microorganism-mediated metabolic pathways and products recently discovered to be associated with HF include trimethylamine (TMA), trimethylamine N-oxidation (TMAO) pathway, short-chain fatty acid (SCFAs) pathway, and the bile acid pathway [9]. The TMA and TMAO pathways play a novel interaction among diet, gut microbiota, atherosclerosis, and thrombosis. TMAO may affect acute myocardial contractility [42,43]. In the process of promoting fermentation, the microbiota in the distal intestine often produces SCFAs, which recognizes specific host receptors to change the host’s blood pressure and lead to hypertension [44]. In addition, there may be other independent mechanisms regulating cardiac function. Primary bile acids have shown to have a direct negative myocardial time-varying effect in a dose-dependent manner [45]. The bile acid receptor-farnesoid X receptor (FXR) can regulate metabolism and inflammation [46], and regulate myocardial cell apoptosis through mitochondrial signaling. Due to the complex mechanism between bile acid and cardiovascular disease-related processes, the potential clinical application of bile acid requires further research.

HF is a result of etiological pathologies by a variety of initial cardiac injuries and subsequent compensatory mechanisms and imbalances in pathogenic processes [47–49]. Inadequate cardiac output during HF often causes intestinal ischemia, edema, and inflammation, which leads to intestinal barrier leakage. This leakage allows inflammatory bacterial products to result in an increase in the bloodstream, further exacerbating inflammation. Furthermore, this leakage changes the intestinal environment. It affects the normal microbial community in the intestine, and the metabolites of these bacteria [9]. Cardiac blood loss decreases during HF, and sympathetic vasoconstriction stimulates adaptive redistribution of the systemic circulation, resulting in congestion of multiple terminal organs, including the intestinal wall [50]. The reduction in perfusion particularly affects the structure of the villi in the intestinal mucosa. Structurally, thickening of the intestinal wall with edema is then observed in patients with HF [51]. It was observed that the intestinal wall thickened with edema in HF patients, and the collagen content in the mucosal wall also increased with the severity of HF. Nonetheless, it is worth noting that thickening of the intestinal wall is directly related to circulation markers of increased levels of C-reactive protein, blood leukocytes, and intestinal permeability. Moreover, in patients with HF, increased circulating levels of multiple cytokines (TNF, IL-1, and IL-6) are associated with more severe and prominent clinical symptoms [52]. The gut microbiota and the cardiovascular system are interdependent. Furthermore, clarifying the complex mechanism between the intestinal and HF, will help pave way for targeted therapeutic strategies for the intestinal flora and HF.

### ICRI and the lungs (pulmonary respiratory organs)

Lungs are one of the organs susceptible to systemic inflammation. When intestinal ICRI occurs, intestinal mucosal barrier is damaged, bacterial translocation, inflammatory factors, endotoxins and other circulating factors, a large number of metabolites and inflammatory mediators continue to accumulate in pulmonary blood vessels and cause lung injury. In addition, endotoxins are considered to be the main cause of acute lung injury (ALI), which induce increased pulmonary endothelial permeability, causing pulmonary edema and ALI [53]. Studies have shown and found that LPS severely damages the pulmonary microvascular endothelium, leading to an acute increase in pulmonary hemagglutinin and protein leakage [54]. Conversely, LPS exposure significantly increases the proinflammatory cytokines TNF-α and IL-1, IL- 6, and IL-8. The expression of the adhesion molecules, increase the number of neutrophils, and these play an important role in the progression of ALI caused by sepsis. These factors then amplify other pro-inflammatory reactions, increase vascular permeability and microvascular leakage, leading to pulmonary edema and injury. In addition, Ferro et al. [55] observed and pin pointed that MPO in neutrophils not only increased their activity in intestinal tissues, but also significantly increased their activity in distant lung organ tissues, which ended up causing local atelectasis.

In acute and chronic lung diseases, inflammatory cytokines and neutrophils produced after lung injury increase microvascular permeability, aggravate lung injury giving rise to metabolites entering and escaping into the blood circulation to reach the intestinal site. The decline of lung function leads to the decrease in blood oxygen content, and the lack of high blood flow in intestinal mucosa cannot maintain excessive oxygen consumption [55]. Henceforth, this further and abruptly damages intestines. On the other hand, both the gut and the lungs trigger the immune response. Dendritic cells of the intestine, respiratory tract, and macrophages in the lung collect antigens in the lumen. Lymphocytes in related lymphoid tissues circulate through the lymphatic system to temper with the systemic immunity. Microbiota also drive pathological changes in intestinal tissues post lung infections [56]. However, more evidence has stated that the effect of intestinal flora on lung immunity, which in turn counterbalances the intestines, is termed the intestinal–lung axis, but its potential pathways and mechanisms are still intensively studied and explored. The lung injury that occurs during ICRI is a complex process involving multiple tissues and organs. These multiple factors are being cross-linked and mutually restricted. However, studies on this phenomenon/concept can provide a theoretical basis for clinical work.

### ICRI and the central nervous system (brain and spinal cord)

Multiple sclerosis (MS) is one of the most common types of central nervous system (CNS) demyelinating disease. The CNS is very sensitive to changes in homeostasis, so it needs its own special barrier, the blood–brain barrier, to maintain normal function. The destruction of the blood–brain barrier is an important marker of the pathophysiology of MS. Immune-mediated disorders of the blood–brain barrier allow activated inflammatory cells to migrate to the brain, leading to demyelination, axon damage, and other tissue damage. Several tight junction molecules in blood–brain barrier endothelial cells are similar to tight junction molecules found within the intestinal tissues, such as occludin, claudins, and zona occludens-1 [57]. Wekerle et al., successfully demonstrated in an experimental model of MS inflammation-autoimmune encephalomyelitis (EAE) and found that the intestinal barrier of patients with MS did change and have some alterations [58]. Ultimately, these changes were at least a part that came up as a result of an intestinal immune response [59,60]. On the contrary, the altered microbiome also causes and gives rise to changes in certain bacteria-related products that tamper with the neuroimmune system. However, inflammation is one of the main reasons for an increased permeability of the intestinal barrier, and the degree of intestinal permeability disorder is closely related to the severity of EAE [61]. Clinical relevance of this result is still unclear and unknown, but there are several possibilities. Among these, one of them is that the intestinal barrier dysfunction is related to the susceptibility to systemic infections [62]. Meanwhile, the CNS and systemic infections are MS and other common complications. In addition, LPS, endogenous interferon, and other MAMPs [37], regulate or maintain nerves by altering the permeability of the intestinal barrier immune disorders. Last but not the least, it is possible that the interaction of the intestinal barrier with the commensal flora modifies the immune response pathologically.

The intestinal barrier regulates multiple biochemical processes and immune regulation of the mucosa. ICRI causes damage to the intestinal mucosal barrier, microbial homeostasis, and the bacteria including corresponding products shift into the blood–brain barrier, causing demyelination of the CNS and MS. LPS is a well-known stimulus of microglia, which can destroy the blood–brain barrier by increasing the production of microglia cells of matrix metalloproteinases [63]. SCFs can directly lead to the structural and immune impairment of microglia cells [64]. SCFAs can also regulate the permeability of the blood–brain barrier. The increase in intestinal permeability leads to the increase in the migration of inflammatory T cells to the CNS, suggesting that it also affects the permeability of the blood–brain barrier [61]. MS therapeutics, in addition to treating the nervous system, the intestinal barrier need to have been improved, including the use of increased tight junctions, increased mucus layers of the intestinal barrier, vitamin D [65], and probiotics [66]. To further understand the pathogenesis of MS, further research and investigations are needed with thorough detailed studies on microbiome, intestinal barrier, and downstream neuroimmunity.

### ICRI and the kidneys

Many studies have found that patients with kidney disease are often accompanied by intestinal flora disorder, and intestinal flora disorder can also accelerate the process of kidney disease [67]. Under physiological conditions, the intestines are sticky membrane has the functions of mechanical barrier, immune barrier, and biological barrier, effectively prevent intestinal bacteria and endotoxin translocation. The level of endotoxin in plasma increased after intestinal congestion and was more significant after reperfusion. Intestinal flora, as a complex microecosystem, plays an important role in human health.

Intestinal bacteria and chronic kidney disease (CKD), the theory of ‘gut–kidney axis’ is put forward by Meijers and Evenepoel, and Pahl and Vaziri [68,69], that is, there is a bidirectional regulation between kidney and colon. In recent years, many scholars have updated it on this basis. Intestinal, kidney, immune relationship, CKD patients will have intestinal flora disorder, and intestinal flora disorder through the bridge of immune response, the effect further aggravated renal injury. With the progression of CKD, the patient’s renal function deteriorates further and eventually enters the stage of irreversible end-stage renal disease (ESRD). In the CKD animal model, the bacteria containing urease convert urea into ammonia, which leads to the destruction of the tight junction (TJ) in the intestinal mechanical barrier, and the tight junction cannot continue to close the gap between adjacent intestinal epithelial cells. Bacteria, endotoxins, microorganisms, antigens, and other substances in the intestinal lumen enter the intestinal mucosa to activate immune cells, leading to abnormal immune responses in the intestinal mucosa. ICRI can also cause the destruction of intestinal tight junctions and the translocation of bacteria and related products. However, when ICRI occurs, the influence of various intestinal flora on the kidney is not very clear. Therefore, more research is needed to reveal the specific mechanism. The improvement of survival time and quality of life of patients with clinically irreversible ESRD provides strong theoretical support.

### ICRI and the IBD

IBDs share a multifactorial etiology of genetic susceptibility, environmental factors, and immune dysregulation. IBD which involves Crohn’s disease (CD) and ulcerative colitis (UC), are characterized by inflammation that compromises the integrity of the epithelial barrier. Both CD and UC share common features such as epithelial breaks, a reduction in tight junction strands, and glandular atrophy [70,71]. For the same reason ICRI has resulted in damage in the intestinal mucosal barrier; due to intestinal mucosal barrier damage and inflammatory response, inflammation-related factors are released into the blood circulation, causing intestinal immune imbalance in the participation of intestinal microbes, resulting in continuous inflammatory damage of intestinal mucosa. TNF is the most important target in the clinical treatment of IBD. Severe IBD may be refractory to steroid and aminosalicylate therapy up to 16% of the time [72]. The use of the anti-TNF drug is critical. Infliximab, adalimumab, and golimumab are effective in refractory disease. Existing experimental studies have shown that ICRI can induce the rat model’s small intestinal mucosal TNF synthesis too. Therefore, is it possible to control ICRI induced IBD by targeting TNF? Overall, a better understanding of the role of ICRI machinery in IBD might aid the design of better therapeutic or preventive strategies for IBDs.

## Conclusions

Based on the complexity of the intestinal structure and morphology, the damage including the disruption caused by intestinal congestion and reperfusion is an intricated pathological process. In spite of local tissue damage, it is also caused by the translocation of various inflammatory factors and bacterial endotoxins. These are always more severe, serious, and life-threatening. Presently, there is limited research progress on ICRI. Therefore, knowledge regarding preclinical research and early clinical findings on ICRI are less investigated upon. Future prospects need to be geared towards more research and investigations on the mechanisms and outcomes of congestion reperfusion injury which are the main focal point and are very essential.

## Abbreviations

CD: Crohn’s disease
CKD: chronic kidney disease
CNS: central nervous system
DAO: diamine oxidase
EAE: autoimmune encephalomyelitis
ESRD: end-stage renal disease
GALT: gut-associated lymphoid tissue
HF: heart failure
IBD: inflammatory bowel disease
ICRI: intestinal congestion reperfusion injury
IL: interleukin
LPS: lipopolysaccharide
MAMP: microbe-associated molecular pattern
MPO: myeloperoxidase
MS: multiple sclerosis
MUC: mucin
PCD: programmed cell death
PMN: polymorphonuclear neutrophil
ROS: reactive oxygen species
SCFA: short-chain fatty acid
sIgA: secretion of immunoglobulin
TLR4: toll-like receptor 4
TMA: trimethylamine
TMAO: trimethylamine N-oxidation
TNF: tumor necrosis factor
TNFR1: TNF-α cell surface death receptor 1
UC: ulcerative colitis
